# Supplementary material for: Microenvironment reconstitution of highly active Ni single atoms on oxygen-incorporated Mo2C for water splitting
Source: Nat Commun. 2024 Feb 13;15:1342. doi: 10.1038/s41467-024-45533-3 (PMC10864306; doi:10.1038/s41467-024-45533-3)
Supplement: Supplementary file 1 — Supplementary Information [file 41467_2024_45533_MOESM1_ESM.pdf]

# Electronic Supplementary Information (ESI)

# Microenvironment Reconstitution of Highly Active Ni Single Atoms on Oxygen-Incorporated $Mo_2C$ for Water Splitting

*Mengyun Hou,[1†] Lirong Zheng,[2†] Di Zhao,[1,*] Xin Tan,[3] Wuyi Feng,[1] Jiantao Fu,[1] Tianxin Wei,[1] Minhua Cao,*[1] Jiatao Zhang,*[1] Chen Chen,*[3]*

[1]*Key Laboratory of Cluster Science, Ministry of Education of China, Beijing Key Laboratory of Photoelectronic/Electrophotonic Conversion Materials, School of Chemistry and Chemical Engineering, Beijing Institute of Technology, Beijing 100081, China*

*E-mail: dizhao@bit.edu.cn; zhangjt@bit.edu.cn; caomh@bit.edu.cn*

[2]*Beijing Synchrotron Radiation Facility, Institute of High Energy Physics, Chinese Academy of Sciences, Beijing 100049, China*

[3]*Engineering Research Center of Advanced Rare Earth Materials, Department of Chemistry, Tsinghua University, Beijing 100084 (China)*

*E-mail: cchen@mail.tsinghua.edu.cn*

† These authors contributed equally to this work.

**Supplementary Figures and Tables**

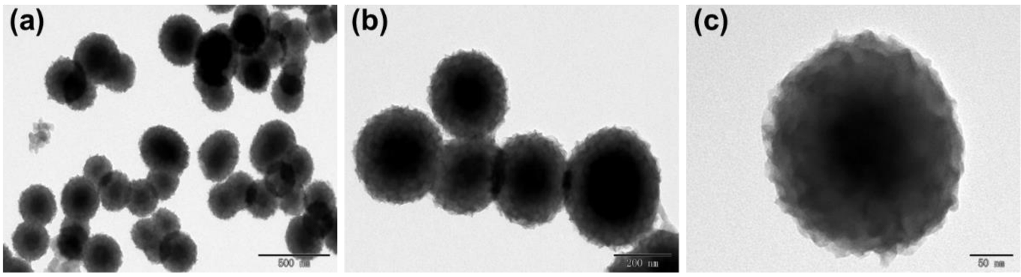


Fig. S1 a-c) TEM images of the amorphous Mo-based precursor.

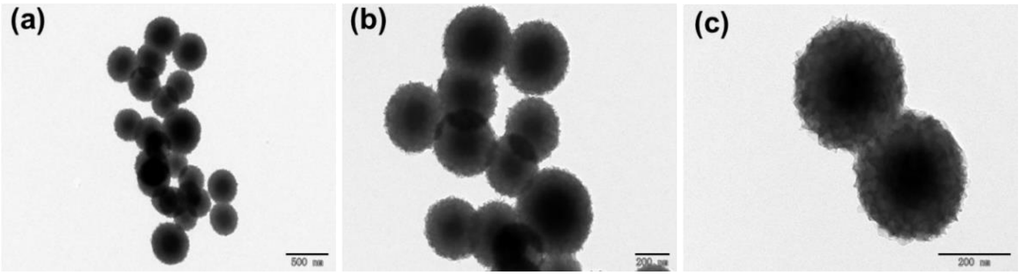


Fig. S2 a-c) TEM image of Mo-based precursor after adsorption of Ni ions.

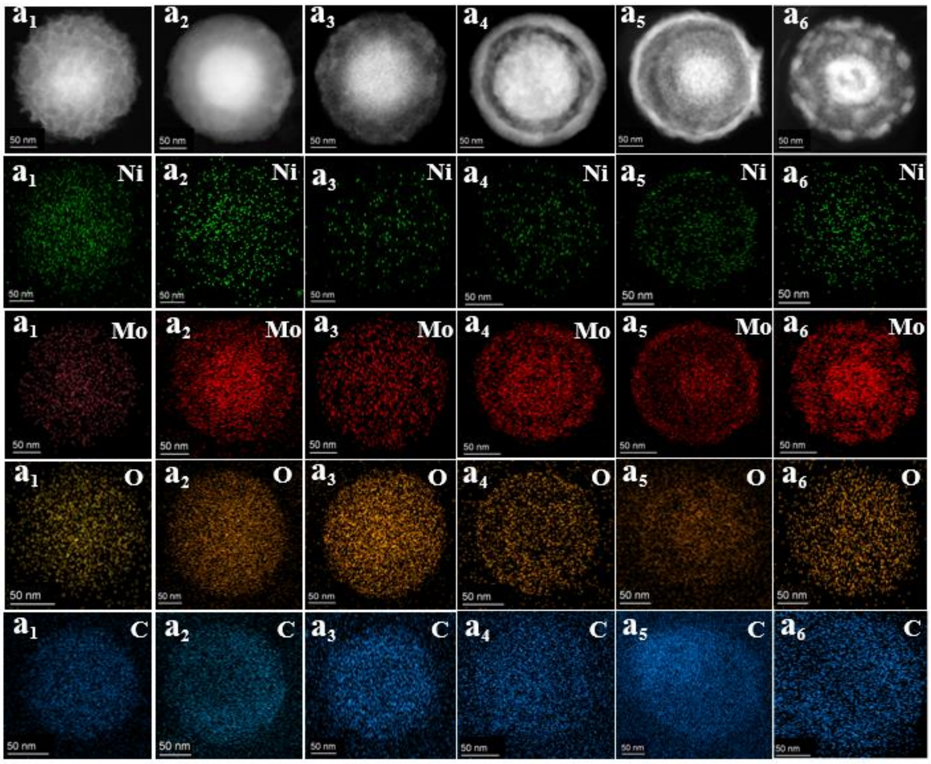


Fig. S3 Comparison of the morphologies and structures of ($a_1$) Mo-based precursor, ($a_2$) $Ni_{SA}$-O/$Mo_2C$-300 ℃ air, ($a_3$) $Ni_{SA}$-O/$Mo_2C$-300 ℃ air - 450 $H_2$/Ar, ($a_4$) $Ni_{SA}$-O/$Mo_2C$-300℃ air -550 $H_2$/Ar，($a_5$) $Ni_{SA}$-O/$Mo_2C$-300℃ air -650 $H_2$/Ar and ($a_6$) the $Ni_{SA}$-O/$Mo_2C$-300℃ air -750 $H_2$/Ar. It mainly presents SAED pattern images and corresponding elemental mapping images of Ni, Mo, O and C elements of all samples,

respectively.

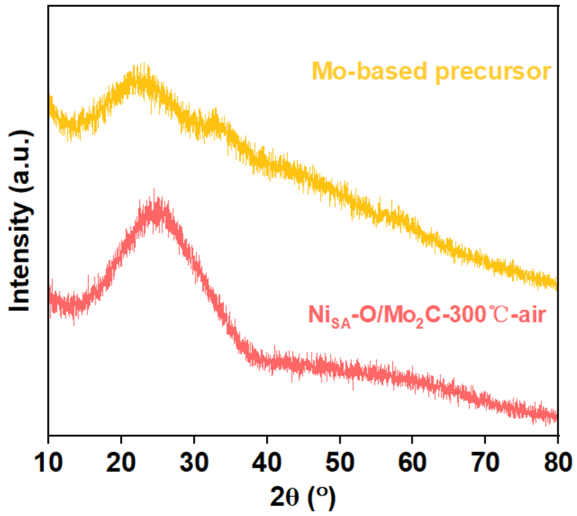


Fig. S4 XRD patterns of Mo-based precursor, $Ni_{SA}$-O/$Mo_2$C-300 ℃ -air which pyrolyzed at 300° in air atmosphere for 1h after adsorption of Ni ions.

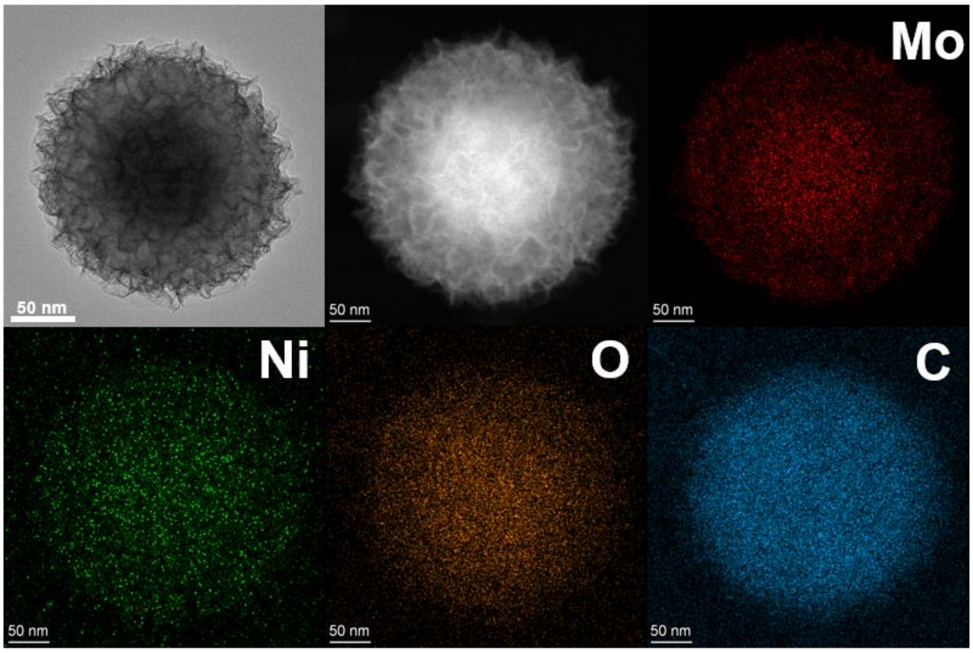


Fig. S5 HR-TEM image, SAED pattern and EDX mapping of Mo, Ni, C and O elements of $Ni_{SA}$-O/$Mo_2$C-$H_2$/Ar.

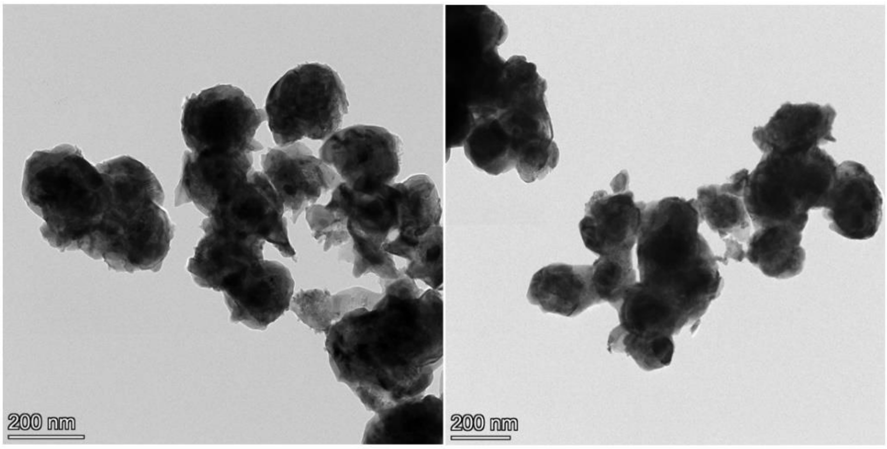


Fig. S6 TEM image of $Ni_{SA}$-O/$Mo_2C$-air-Ar：Pyrolysis at 300° for 1h in air atmosphere, followed by 750 °C for 3 h under Argon atmosphere with with a heating rate of 2 °C.

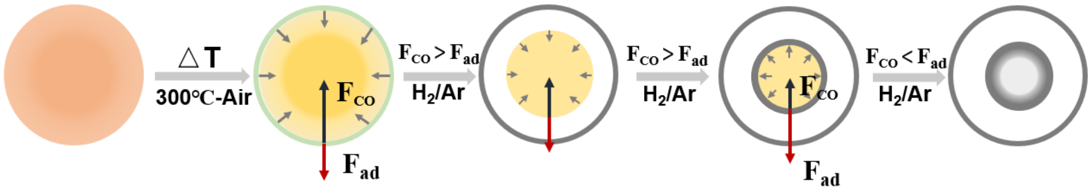


Fig. S7 Schematic illustration of the fabrication of double-shelled $Ni_{SA}$-O/$Mo_2C$ hollow nanosphere.

The evolution of pyrolysis intermediates along with temperature was then tracked via TEM and corresponding elemental mapping of Mo, Ni, O, C. We selected four pyrolysis intermediates to monitor the evolution of structures: low temperature calcination at 300 ℃ in air; 300 °C in air followed by 450 °C; 300 ℃ in air followed by 550 °C and 300 °C in air followed by 650 °C for 3 h under hydrogen-argon ($H_2$/Ar) atmosphere, respectively. Combined with the original Mo-based organic precursors and the main sample obtained under the condition of 300 °C in air followed by 750 for 3 h, we made a comparison of Fig. S3. It was noting that the double-shelled hollow structure of $Ni_{SA}$-O/$Mo_2C$ was obtained at certain temperatures under air and sequential $H_2$/Ar atmospheres. From SAED pattern images ($a_1$-$a_6$), all of them well maintain the morphology of nanosphere of the precursor. Mo, Ni and O elements almost have the same shape and C elements is uniformly distributed in an individual $Ni_{SA}$-O/$Mo_2C$ nanospheres. The original Mo-based organic precursors are sold. After calcination at 300 ℃in air ($a_2$), the intermediate shows an fuzzy core-shell structure, in which Mo and Ni elements aggregate towards the core. Meanwhile, after XRD tests, the weakly crystalline of Mo-based organic precursors transform to amorphous $MoO_x$-related material (Fig. S4), which can be proved by the more obvious O element mapping of intermediate $a_2$. Subsequently, with the elevation of pyrolysis temperatures in $H_2$/Ar atmosphere, the obvious core-shell structure is formed. Then the core become smaller and smaller, until double shelled $Ni_{SA}$-O/$Mo_2C$ hollow nanosphere is gradually formed.

The distribution of Mo element is completely consistent with the structural changes. In order to verify the roles of air and $H_2$/Ar atmospheres. We first calcined the Mo-based organic precursors with only $H_2$/Ar atmospheres. As shown in Fig. S5, there is no significant changes between the shape of the calcined sample and the precursor, which means that the first step of air calcination is very important in the formation of double-shelled hollow structure. Then, we calcined the Mo-based organic precursors with air and subsequent Ar atmospheres. As shown in Fig. S6, although there is a small amount of core-shell structure, there is no double-shelled hollow structure and the spherical shape of precursor is not maintained very well. Based on all the results above, we conclude the formation of double-shelled hollow structure is mainly based on the early oxidation from air and non-equilibrium non-uniform shrinkage caused by heat treatment. The schematic illustration is shown in Fig. S7. At the initial stage of calcination in air, there exists an early oxidation from air and meanwhile a large temperature gradient ($\Delta$T) along the radical direction, which leads to the formation of a $MoO_x$ and $MoO_y$ fuzzy core-shell structure. Two forces of opposite directions (the contraction force: $F_{co}$; the adhesion force:$F_{ad}$) act on the interface between the $MoO_x$ shell and the $MoO_y$ core (the so-called heterogeneous contraction). Subsequently, during the calcination in $H_2$/Ar atmospheres, when $F_{co}$ is larger than the $F_{ad}$, the larger $F_{co}$ causes the inner core to shrink inward, and the $F_{ad}$ of the shell prevents it from shrinking inward, eventually resulting in the core shrinking and separating from the shell. With the increase of heating temperature, $F_{co}$ decreases continuously, and when $F_{ad}$ exceeds $F_{co}$, the direction of material movement will be reversed. Eventually, the core shrinks outward, leaving a hole in the core. Based on this formation mechanism, the step of air calcination is conducive to the formation of amorphous molybdenum oxide. The $H_2$/Ar atmosphere helps to form $Mo_2C$ rigid shell that generates $F_{ad}$ and also is conducive to the volatilization of internal substances and enhance $F_{co}$.

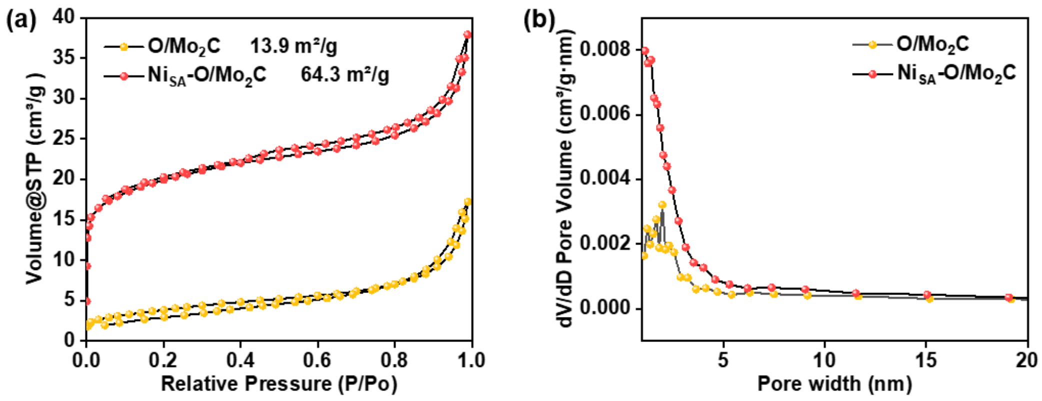


Fig. S8 a) $N_2$ adsorption/desorption isotherms. b) pore size distribution curves calculated from the adsorption branch of the isotherms of $O/Mo_2C$ and $Ni_{SA}$-$O/Mo_2C$.

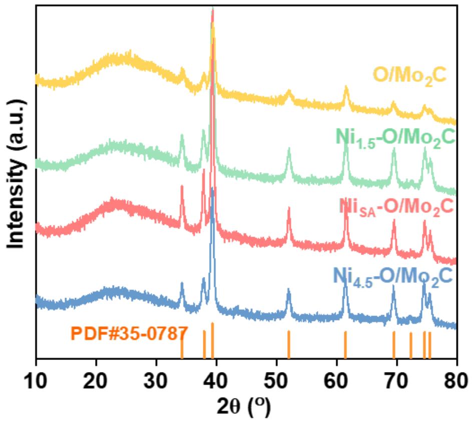


Fig. S9 XRD patterns of $O/Mo_2C$ and electrocatalysts obtained at different Ni doping.

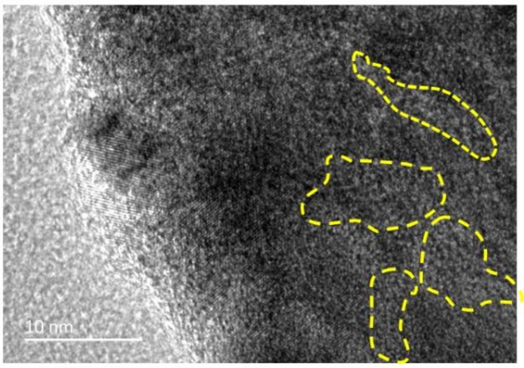


Fig. S10 HR-TEM images of the $Ni_{SA}$-$O/Mo_2C$, the carbon substrate is observed in the lighter regions of the surface of sphere (highlighted by yellow circles), while the darker regions are $Mo_2C$ nanocrystals with lattice stripes.

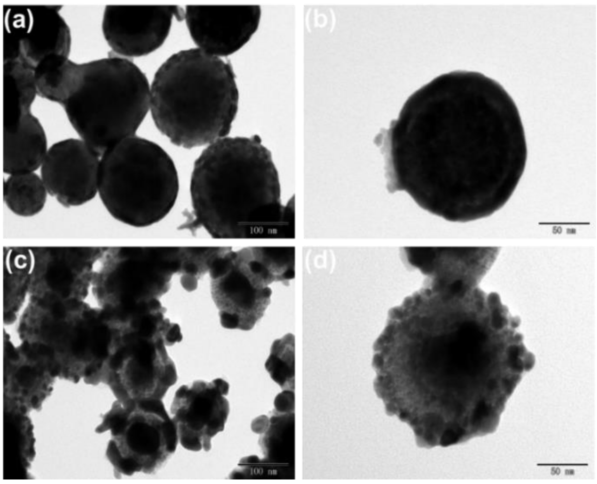


Fig. S11 a-b) TEM images of the $Ni_{1.5}$-$O/Mo_2C$. c-d) $Ni_{4.5}$-$O/Mo_2C$.

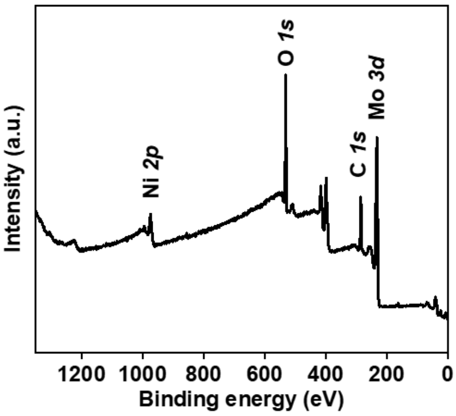


Fig. S12 The survey spectrum of $Ni_{SA}$-O/$Mo_2C$.

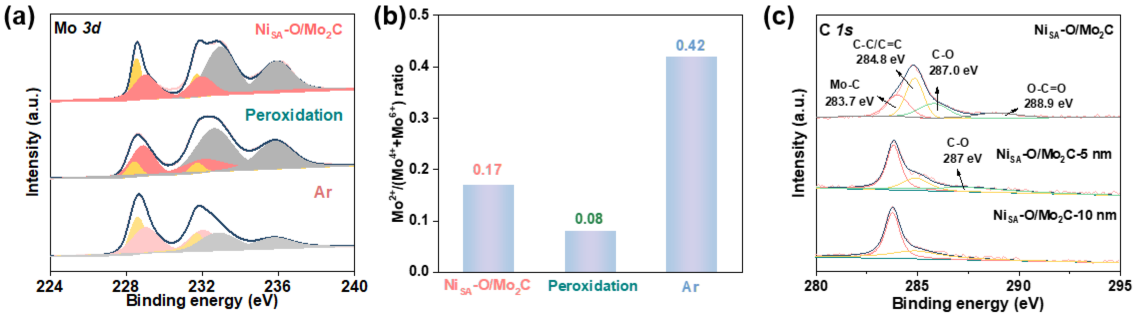


Fig. S13 (a) High-resolution XPS spectra for Mo *3d*. The $Ni_{SA}$-O/$Mo_2C$ was taken it out from tubular furnace after calcination at natural cooling to 30 °C. The peroxidation $Ni_{SA}$-O/$Mo_2C$ sample was taken it out at 60 °C. The $Ni_{SA}$-O/$Mo_2C$-Ar was naturally cooled to room temperature and then taken it out after protecting it in Ar gas for 12 h; (b) The area ratio of $Mo^{2+}$/($Mo^{4+}$+$Mo^{6+}$) from XPS; (c) High-resolution XPS spectra for C *1s* with an etching depth of 5 nm and 10 nm, respectively.

The process of taking out the samples after pyrolysis can be adjusted to control the degree of oxidation of $Mo_2C$. The low oxidation state of $Mo^{2+}$ in the newly reduced $Ni_{SA}$-O/$Mo_2C$ is easy to be oxidized. Based on this, we proposed two states of peroxidation and lower oxidation. For the $Ni_{SA}$-O/$Mo_2C$, we take out from the tube furnace and exposed to the air at 30 °C. Wihle we take the newly reduced $Ni_{SA}$-O/$Mo_2C$ out of the tube furnace and expose to the air at 60 °C to get the peroxidized sample. To get sample with lower oxidation, we saved the newly reduced $Ni_{SA}$-O/$Mo_2C$ in Ar for 12 h. As shown in Fig. S13a, after the newly reduced $Ni_{SA}$-O/$Mo_2C$ was treated with Ar environment for 12 h, the proportion of $Mo^{2+}$ increased significantly, while the proportion of high oxidation state of $Mo^{4+}$ and $Mo^{6+}$ obviously decreased. In the peroxidized sample, the proportion of $Mo^{2+}$ decreases obviously while the proportion of $Mo^{4+}$ and $Mo^{6+}$ increases significantly. We further calculated their area ratios of $Mo^{2+}$ to high oxidation state $Mo^{4+}$ and $Mo^{6+}$ from XPS fitting results (Fig. S13b). The results show that the ratio is only 0.08 after peroxidation, and 0.46 after Ar environment

through inert treatment, indicating that the $Mo_2C$ surface has a strong oxidation behavior when exposed to air at high temperature, while the Ar environment treatment prevented the oxidation behavior of $Mo^{2+}$. In other words, the $Mo_2C$ oxide layer comes from the oxidation of air, and different temperatures affect the intensity of the oxidation behavior. So, controlling the temperature at which the sample is taken from the tubular furnace and controlling the condition in which the sample is protected by an inert gas can control the level of surface oxidation of $Mo_2C$.

We further performed XPS at different etching depths of 5 nm and 10 nm to explore the surface oxidation degree of the $Ni_{SA}$-O/$Mo_2C$ samples. Fig. 2a show that the proportion of high oxidation state $Mo^{4+}$ and $Mo^{6+}$ decreases significantly with the increase of etching depth. At the etching depth of 5 nm, only a small amount of $Mo^{6+}$ exists. However, there is basically only $Mo^{2+}$ at the depth of 10 nm. At the same time, it can be observed from the XPS spectrum of C1s that Mo-C gradually increased with inward etching (Fig. S13c). All these results prove that the predominant phase of $Ni_{SA}$-O/$Mo_2C$ is $Mo_2C$, which surface is decorated by partially oxidized $MoO_x$.

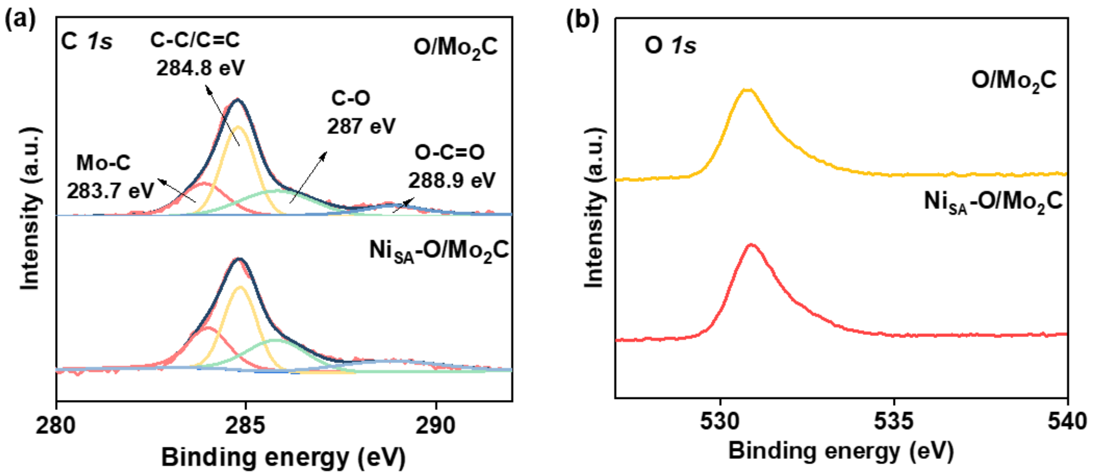


Fig. S14 (a) C *1s* XPS spectra of O/$Mo_2C$ and $Ni_{SA}$-O/$Mo_2C$; (b) O *1s* XPS spectra of O/$Mo_2C$ and $Ni_{SA}$-O/$Mo_2C$, respectively.

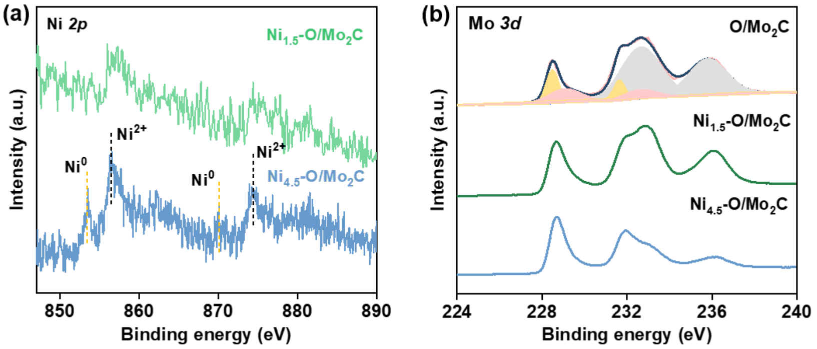


Fig. S15 (a) Ni *2p* XPS spectra of $Ni_{1.5}$-O/$Mo_2C$ and $Ni_{4.5}$-O/$Mo_2C$ catalyst; (b) Mo *3d*

XPS spectra of $O/Mo_2C$, $Ni_{1.5}$-$O/Mo_2C$ and $Ni_{4.5}$-$O/Mo_2C$, respectively.

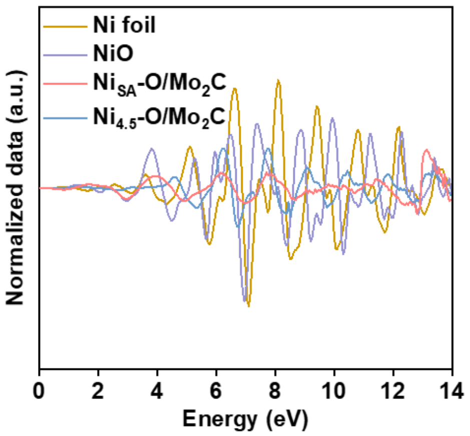


Fig. S16 k space EXAFS curves of $Ni_{SA}$-$O/Mo_2C$, Ni foil, NiO and $Ni_{4.5}$-$O/Mo_2C$.

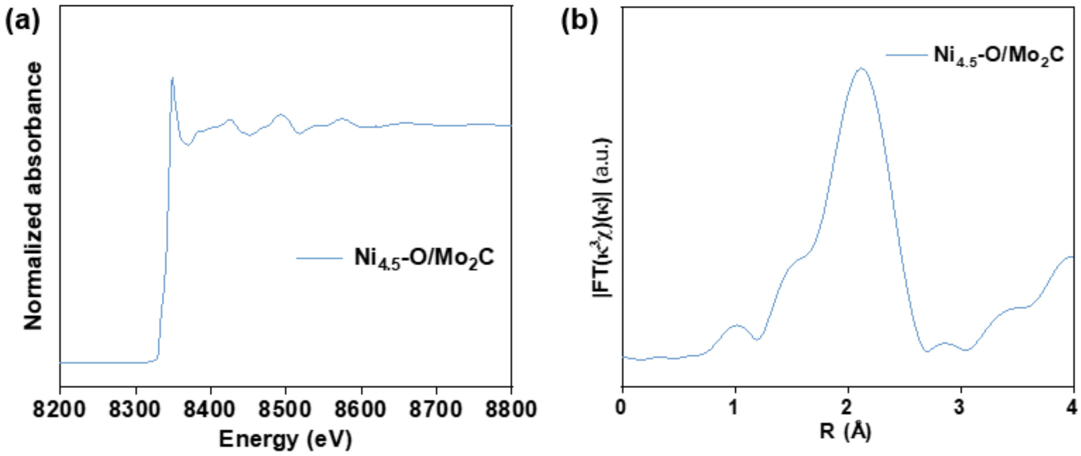


Fig. S17 a) Mo K-edge XANES spectra; b) EXAFS FT $k^3$ -weighted $\chi(k)$ function spectra of $Ni_{4.5}$-$O/Mo_2C$.

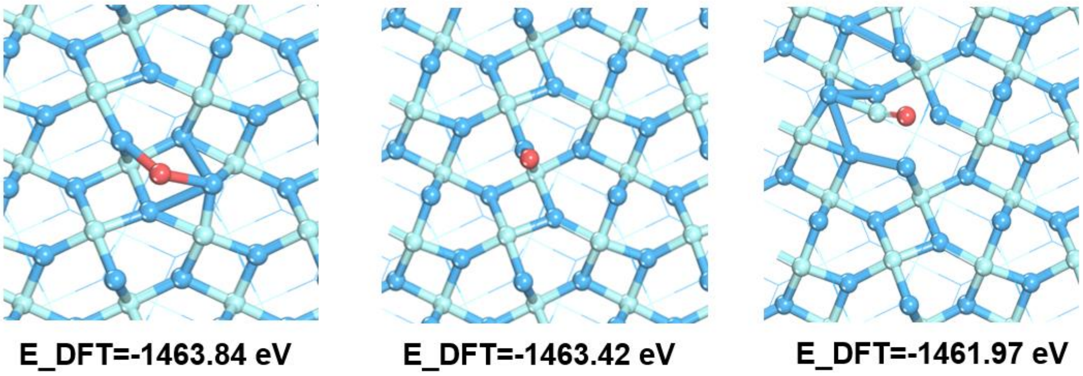


Fig. S18 The plots of different optimal adsorption structures for oxygen on $O/Mo_2C$ facet and several total energies (E_DFT).

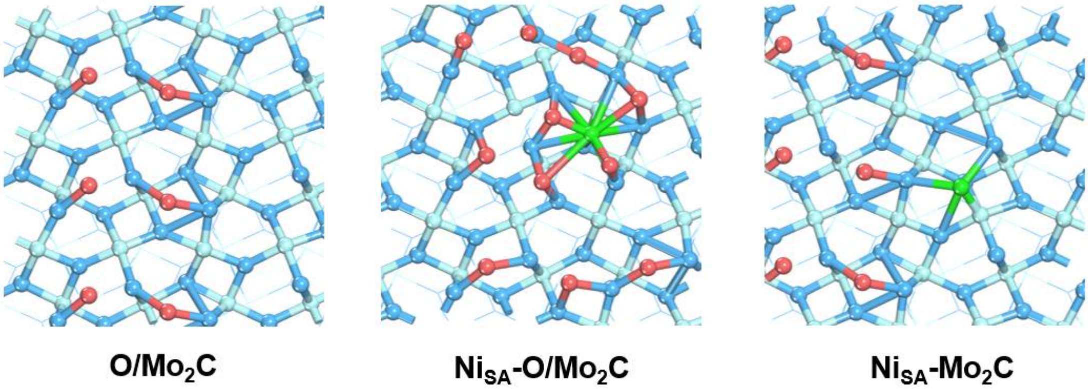


Fig. S19 The plots of partially oxidized facet of $Mo_2C$ ($O/Mo_2C$) and different optimal adsorption structures for nickel ($Ni_{SA}$-$O/Mo_2C$ and $Ni_{SA}$-$Mo_2C$).

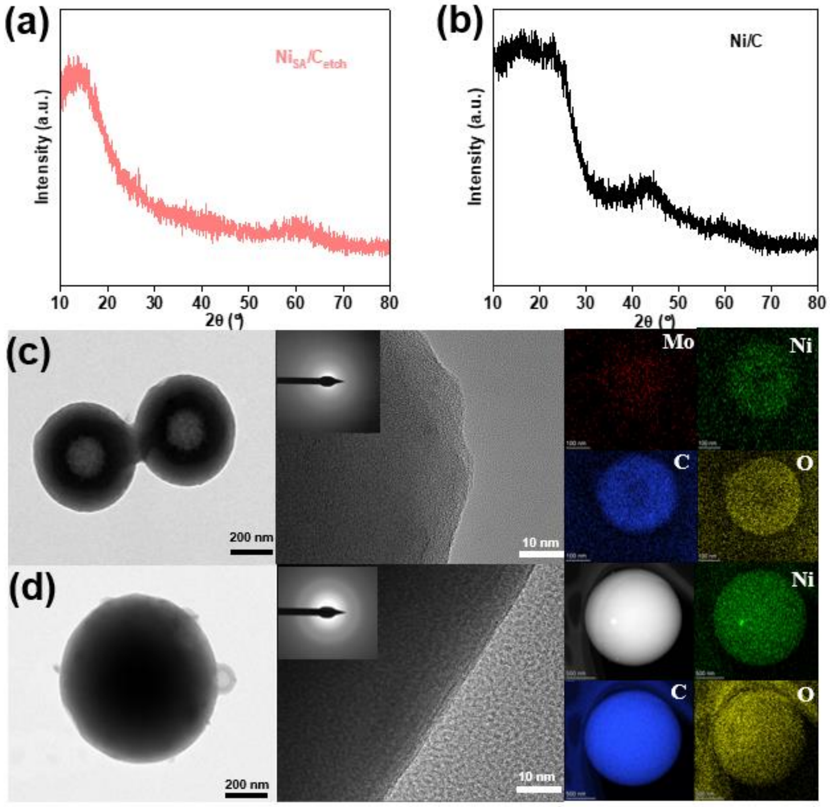


Fig. S20 (a, b) XRD patterns of $Ni_{SA}/C_{etch}$ and Ni/C; (c, d) HR-TEM image of $Ni_{SA}/C_{etch}$ and Ni/C, respectively. Insets in (c): SAED pattern, and Elemental mapping of Ni, O, C and Mo for an individual nanospheres.

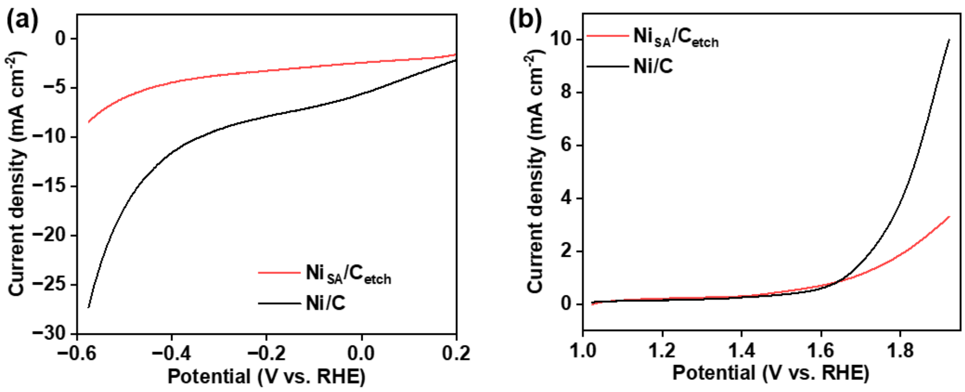


Fig. S21 (a-b) Polarization curves of Ni/C and $Ni_{SA}/C_{etch}$ for HER and OER.

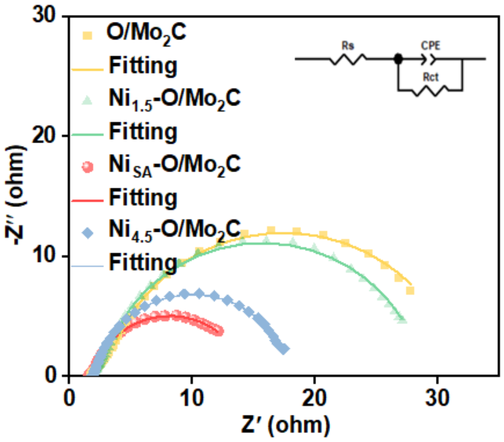


Fig. S22 The electrochemical impedance spectroscopy (EIS) of the catalysts. The Nyquist plots of $O/Mo_2C$ and $Ni_{SA}$-$O/Mo_2C$, $Ni_{1.5}$-$O/Mo_2C$, $Ni_{4.5}$-$O/Mo_2C$.

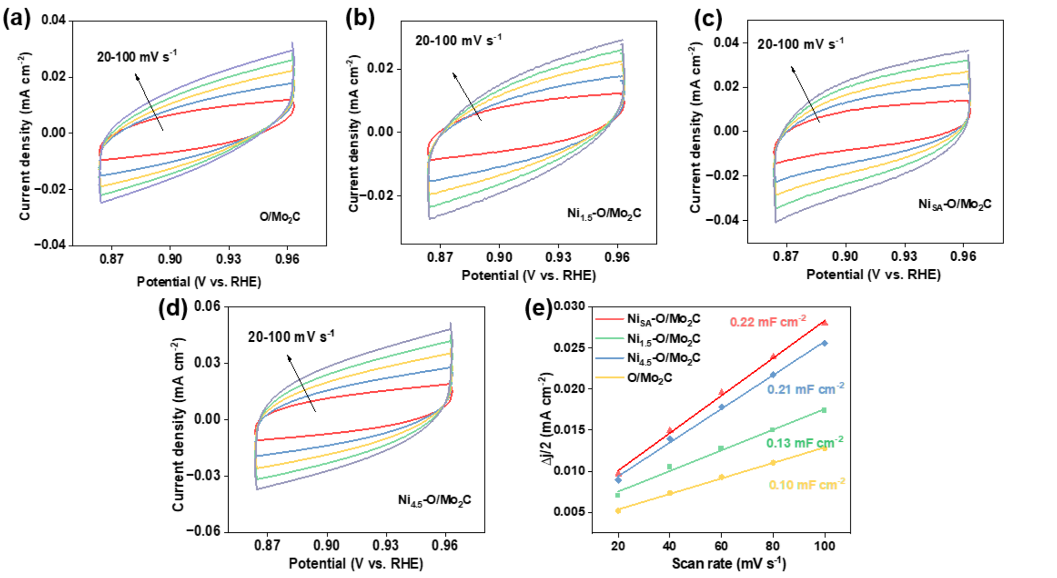


Fig. S23 CVs were performed at various scan rates of 20, 40, 60, 80, and 100 mV $s^{-1}$: a) $O/Mo_2C$, b) $Ni_{1.5}$-$O/Mo_2C$; c) $Ni_{SA}$-$O/Mo_2C$, d) $Ni_{4.5}$-$O/Mo_2C$. e) Electrochemical active surface areas of $O/Mo_2C$ and $Ni_{SA}$-$O/Mo_2C$, $Ni_{1.5}$-$O/Mo_2C$, $Ni_{4.5}$-$O/Mo_2C$.

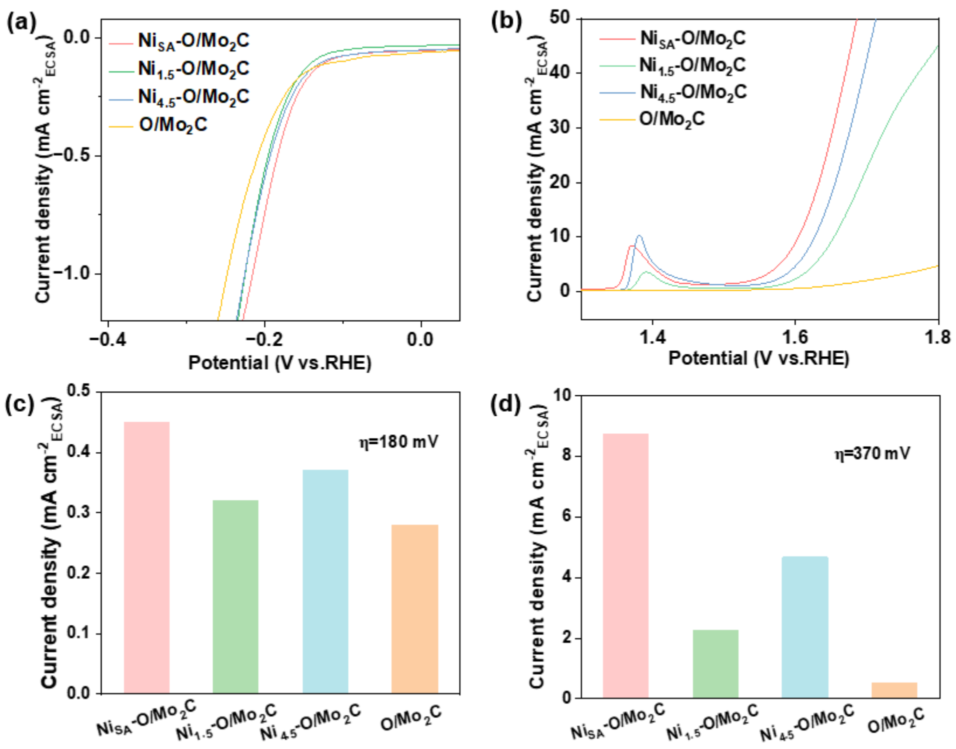


Fig. S24 (a-b) Polarization curves of $Ni_{SA}$-O/$Mo_2C$, $Ni_{1.5}$-O/$Mo_2C$, $Ni_{4.5}$-O/$Mo_2C$ and O/$Mo_2C$ for HER and OER normalized by ECSA. (c-d) Comparison of HER, OER specific activity of $Ni_{SA}$-O/$Mo_2C$, $Ni_{1.5}$-O/$Mo_2C$, $Ni_{4.5}$-O/$Mo_2C$ and O/$Mo_2C$ at 180 mV, 370 mV overpotential through polarization curves normalized by ECSA, respectively.

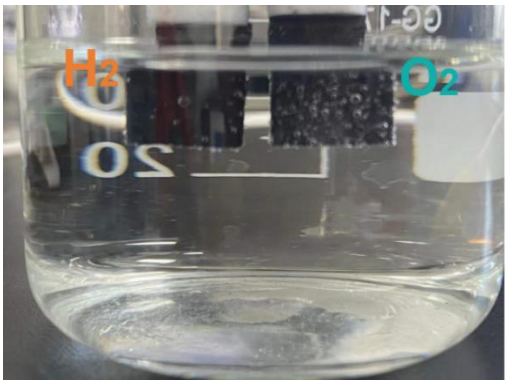


Fig. S25 Demonstration of the evolution of $H_2$ and $O_2$ on $Ni_{SA}$-O/$Mo_2C$||$Ni_{SA}$-O/$Mo_2C$.

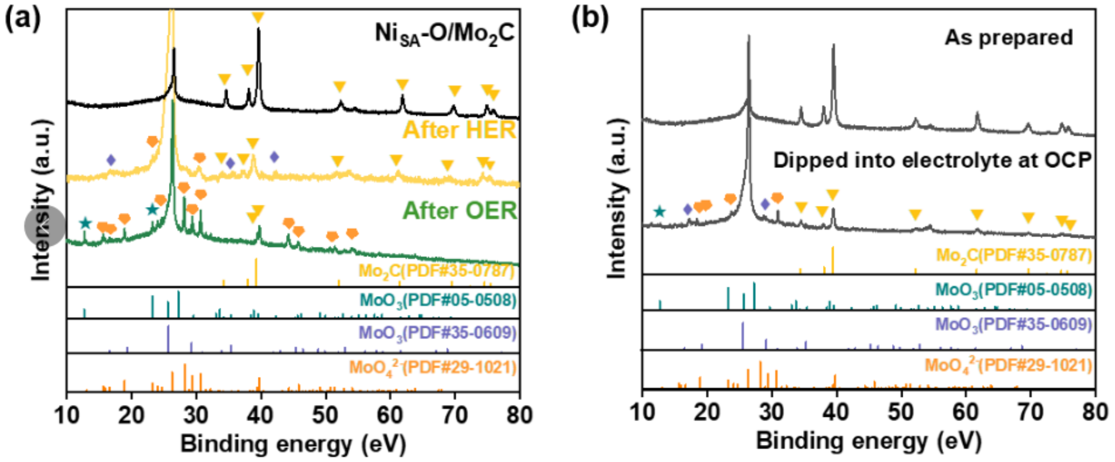


Fig. S26 XRD characterization of $Ni_{SA}$-O/$Mo_2C$ catalyst (a) after HER and OER test; (b) as prepared/dipped into the KOH electrolyte at OCP.

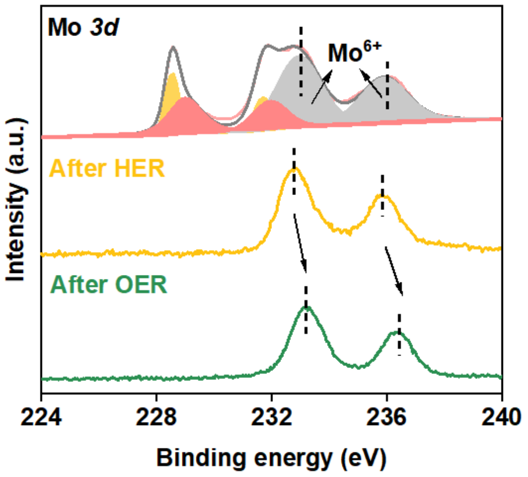


Fig. S27 XPS spectra of Mo *3d* for $Ni_{SA}$-O/$Mo_2C$ before and after HER and OER stability test.

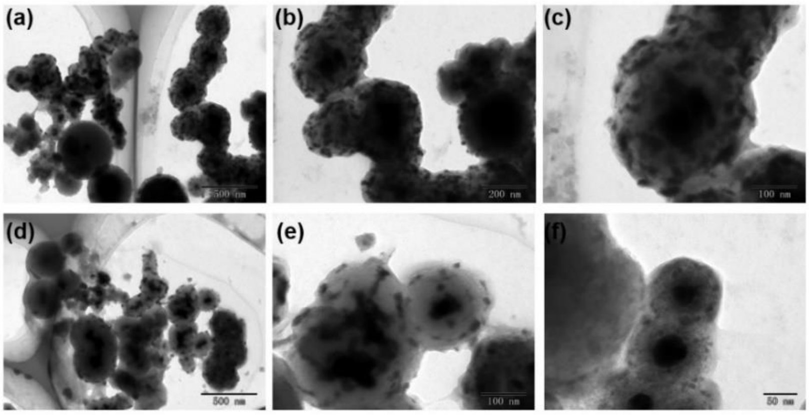


Fig. S28 a-c) TEM image of $Ni_{SA}$-O/$Mo_2C$ after HER and d-f) OER electrolysis in 1 M KOH.

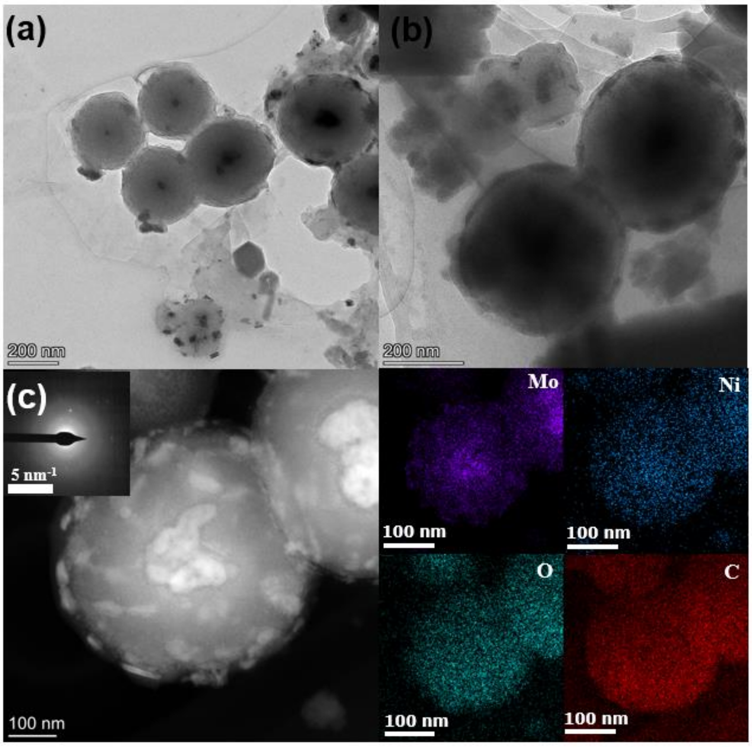


Fig. S29 a-b）HRTEM image; c-d) STEM and EDS element mapping of Mo, Ni, O, C of the $Ni_{SA}$-O/$Mo_2$C catalysts after HER. Inset in (c) shows selected area electron diffraction (SAED) pattern.

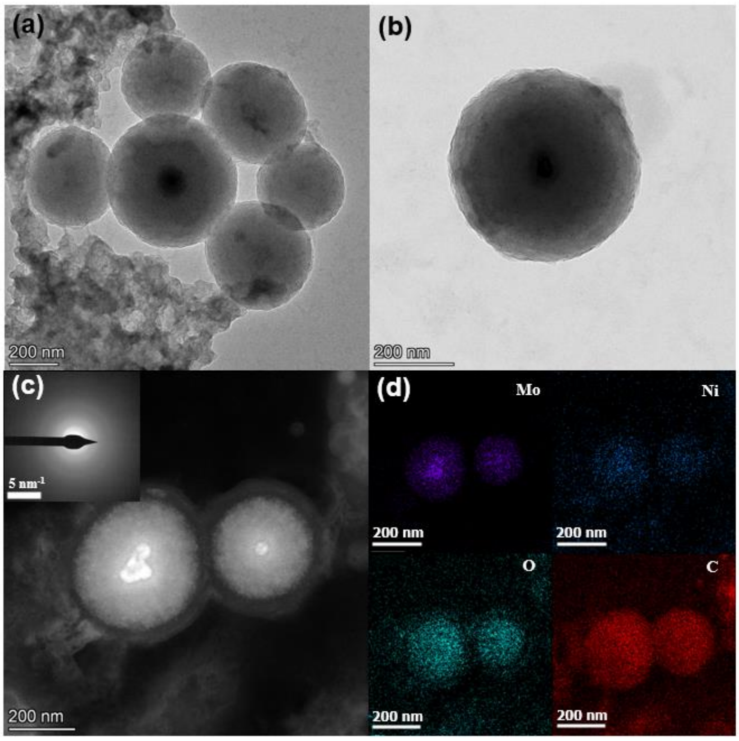


Fig. S30 a-b）HRTEM image; c-d) STEM and EDS element mapping of Mo, Ni, O, C of the $Ni_{SA}$-O/$Mo_2$C catalysts after OER. Inset in (c) shows SAED pattern.

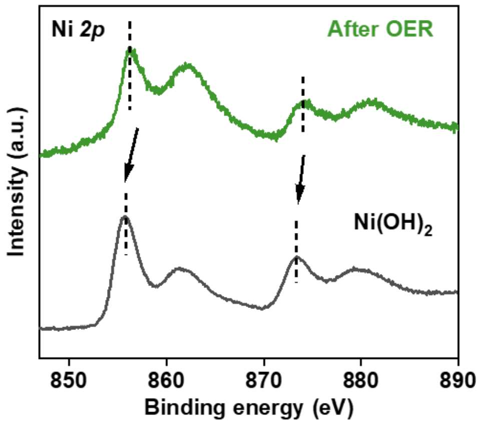


Fig. S31 Ni *2p* XPS spectra of $Ni(OH)_2$ and $Ni_{SA}$-O/$Mo_2C$ after OER durability test.

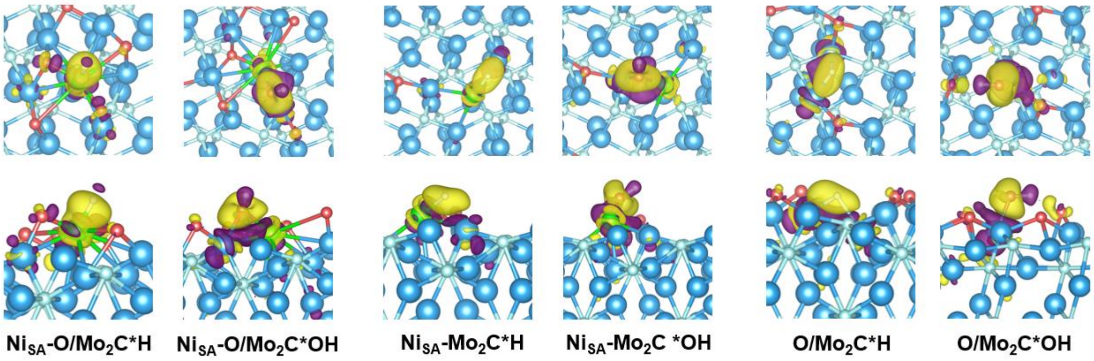


Fig. S32 The plots of charge density differences with $Ni_{SA}$-O/$Mo_2C$, $Ni_{SA}$-$Mo_2C$ and O/$Mo_2C$, are, from top to down: top view of 3D plot and side view. The isosurface level set to 0.003 $eÅ^{-3}$, where charge depletion and accumulation were depicted by purple and yellow, respectively.

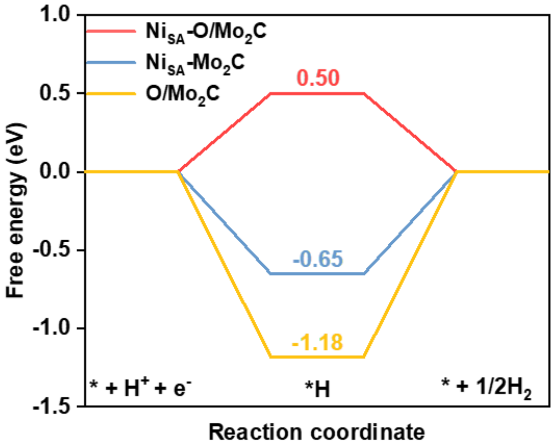


Fig. S33 Gibbs free energy change of HER pathways on Ni sites of $Ni_{SA}$-O/$Mo_2C$, $Ni_{SA}$-$Mo_2C$ and O/$Mo_2C$.

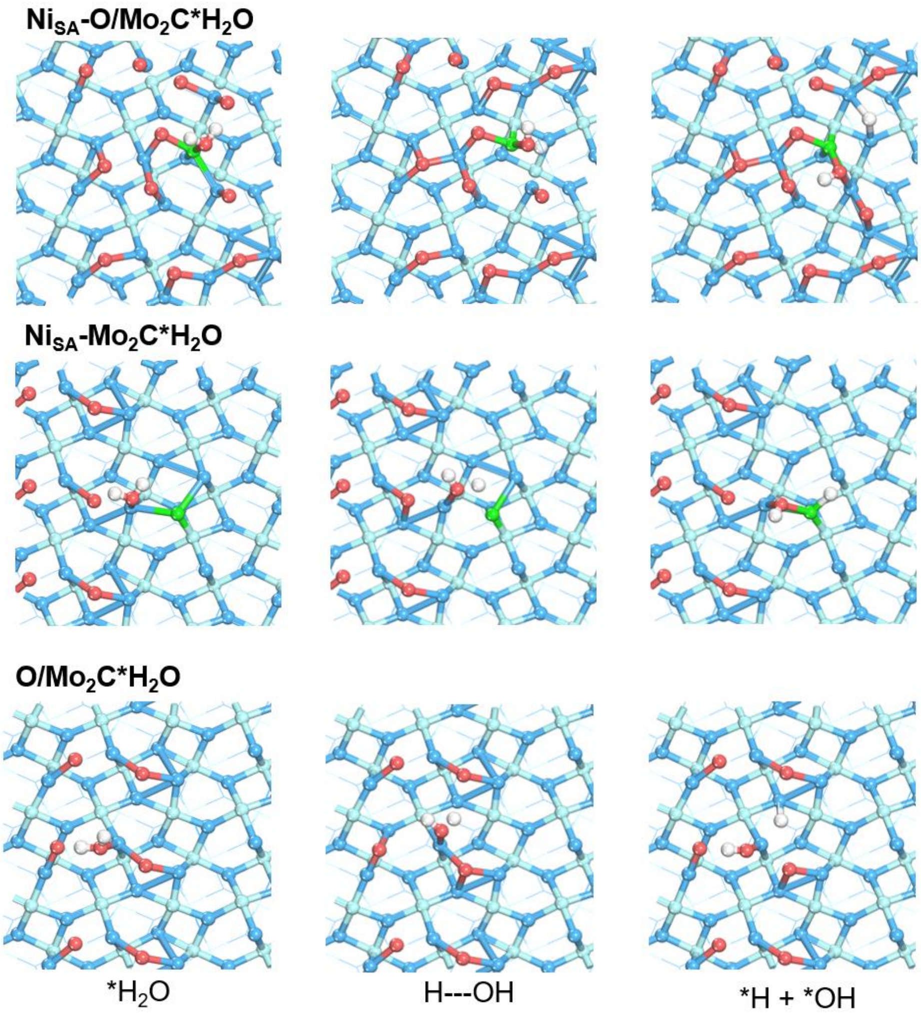

Fig. S34 Diagram of the optimal models for Volmer step at $Ni_{SA}$-O/$Mo_2C$ and $Ni_{SA}$-$Mo_2C$, O/$Mo_2C$.

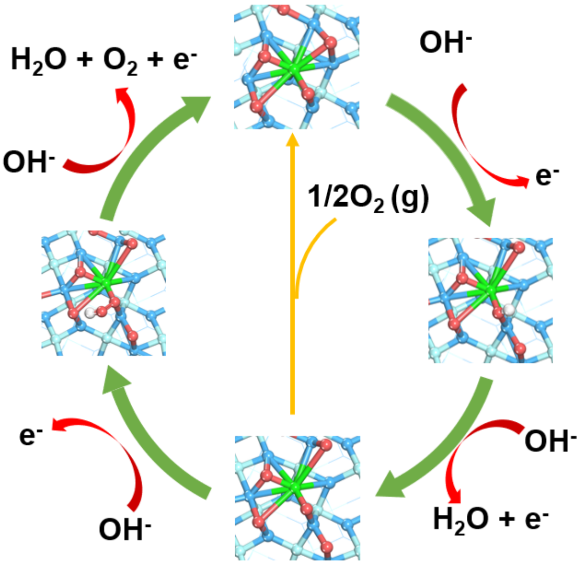

Fig. S35 Diagram of the optimal models for OER reaction step at Ni site of $Ni_{SA}$-O/$Mo_2C$.

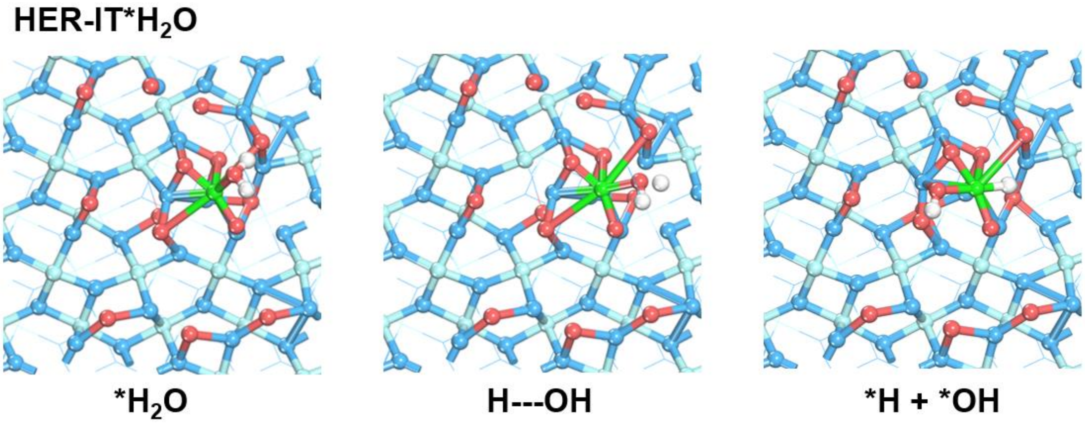


Fig. S36 Diagram of the optimal models for Volmer step at $Ni_{SA}$-$MoO_x$/$Mo_2C$ after HER-IT.

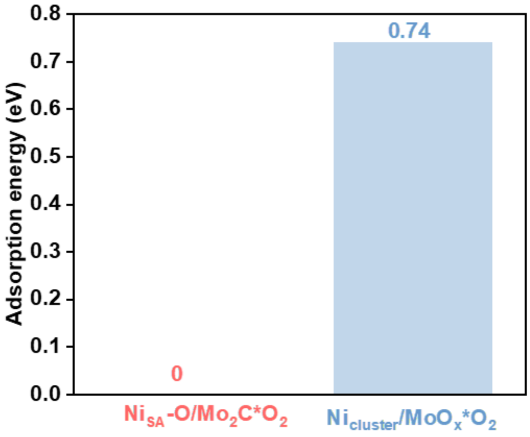


Fig. S37 The chemisorption energies of $O_2$ of $Ni_{SA}$-O/$Mo_2C$ and $Ni_{cluster}$/$MoO_x$.

**Table S1** Mo, Ni content calculated by ICP-OES results.

| Sample | Mo | Ni |
|---|---|---|
| | **wt %** | **wt %** |
| **$Ni_{1.5}$-O/$Mo_2C$** | 86.59 | 0.75 |
| **$Ni_{SA}$-O/$Mo_2C$** | 88.91 | 0.98 |
| **$Ni_{4.5}$-O/$Mo_2C$** | 97.49 | 1.33 |

**Table S2** The content of each element in the $Ni_{SA}$-O/$Mo_2C$ calculated by XPS.

| Name | Mo | Ni | C | O |
|---|---|---|---|---|
| **Atomic (%)** | 10.08 | 0.91 | 45.9 | 15 |

**Table S3** Structural parameters of $Ni_{SA}$-O/$Mo_2C$ extracted from the EXAFS fitting. ($S0^2$=0.80)

| Sample | Scattering pair | CN | R(Å) | $\sigma^2(10^{-3}Å^2)$ | $\Delta E_0$(eV) |
|---|---|---|---|---|---|
| **$Ni_{SA}$-O/$Mo_2C$** | Ni-O | 3.9±0.4 | 2.01±0.02 | 5.6±0.5 | 3.4±0.6 |
| | Ni-Mo | 5.0±0.8 | 2.98±0.02 | 6.9±0.6 | -5.3±0.5 |

$S_0^2$ is the amplitude reduction factor $S_0^2$=0.8; CN is the coordination number; R is interatomic distance (the bond length between central atoms and surrounding coordination atoms); $\sigma^2$ is Debye-Waller factor (a measure of thermal and static disorder in absorber-scatterer distances); $\Delta E_0$ is edge-energy shift (the difference between the zero kinetic energy value of the sample and that of the theoretical model). R factor is used to value the goodness of the fitting.

**Table S4** Comparison of OER performance of $Ni_{SA}$-O/$Mo_2C$ and other reported non-precious metal single-atom electrocatalysts.

| Electrocatalysts | Electrolyte | $\eta_{10}$ (mV vs. RHE) | Tafel slope (mV dec$^{-1}$) | Working electrode | Ref |
|---|---|---|---|---|---|
| **$Ni_{SA}$-O/$Mo_2C$** | 1.0 M KOH | 299 | 89 | carbon paper | This work |
| **Co1/$TaS_2$** | 1.0 M KOH | 330 | 70 | glassy-carbon | ACS Nano. **2021**, 15, 7105−7113. |
| **NiPc-GO** | 1.0 M KOH | 320 | 61 | glassy-carbon | ACS Nano. **2020**, 14, 13279-13293. |
| **HCM@Ni-N** | 1.0 M KOH | 304 | 76 | carbon paper | Adv. Mater. **2019**, 31, 1904548. |
| **CoNi-SAs/NC** | 1.0 M KOH | 340 | 58.7 | carbon cloth | Adv. Mater. **2019**, 31, 1905622. |
| **$NiSO_4$-GF** | 1.0 M KOH | 300 | 80 | graphite foil | ACS Nano. **2020**, 14, 11662-11669. |

| | | | | | |
|---|---|---|---|---|---|
| **NiFe-CNG** | 1.0 M KOH | 270 | 74 | glassy-carbon | Nat. Commun. **2021**, 12, 1-13. |
| **Co SAs/$Mo_2C$** | 1.0 M KOH | 270 | 74.9 | carbon cloth | J. Mater. Chem. A,**2020**, 8, 3071–3082. |
| **Ni-NHGF** | 1.0 M KOH | 331 | 63 | glassy-carbon | Nat. Catal. **2018**, 1, 63- 72. |
| **Co-NHGF** | 1.0 M KOH | 402 | 80 | glassy-carbon | Nat. Catal. **2018**, 1, 63- 72. |
| **Fe-NHGF** | 1.0 M KOH | 488 | 175 | glassy-carbon | Nat. Catal. **2018**, 1, 63- 72. |
| **Co-POC** | 0.1 M KOH | 470 | 139 | glassy-carbon | Adv. Mater. **2019**, 31, 1900592. |
| **Fe/N-G-SAC** | 0.1 M KOH | 370 | 73 | glassy-carbon | Adv. Mater. **2020**, 32, 2004900. |
| **Fe-$N_4$ SAs/NPC** | 1.0 M KOH | 430 | 95 | glassy-carbon | Angew. Chem., Int. Ed., **2018,** 57, 8614–8618 |
| **CoSA/N,S-HCS** | 1.0 M KOH | 306 | 38 | glassy-carbon | Adv. Energy Mater. **2020**, 10, 2002896 |
| **Ni SAs@S/N-CMF** | 1.0 M KOH | 285 | 50.8 | carbon paper | Adv. Mater. 2203442. |
| **Ni-N,P/CNFs** | 1.0 M KOH | 330 | 65 | carbon cloth | Nano Energy, 2022, 98， 107266. |
| **Ni-N,S/CNFs** | 1.0 M KOH | 350 | 111 | carbon cloth | Nano Energy, 2022, 98: 107266. |
| **CoSAs-$MoS_2$/TiN NRs** | 1.0 M KOH | 340.6 | 81.2 | carbon cloth | Adv Funct Mater, 2021, 31,2100233. |

**Table S5** Comparison of HER performance for $Ni_{SA}$-O/$Mo_2C$ single-atom catalysts and non-precious metal single-atom catalysts reported in the literature.

| Electrocatalysts | Electrolyte | $\eta_{10}$ (mV vs. RHE) | Tafel slope (mV $dec^{-1}$) | Working electrode | Ref |
|---|---|---|---|---|---|
| **$Ni_{SA}$-O/$Mo_2C$** | 1.0 M KOH | 133 | 83.6 | glassy-carbon | This work |

| | | | | | |
|---|---|---|---|---|---|
| **CoSA/N,S-HCS** | 1.0 M KOH | 165 | 96 | glassy-carbon | Adv. Energy Mater. **2020**, 10, 2002896 |
| **$Mo_1N_1C_2$** | 0.1 M KOH | 132 | 90 | glassy-carbon | Angew. Chem., Int. Ed., **2017**, 56, 16086–16090 |
| **$Fe-N_4$ SAs/NPC** | 1.0 M KOH | 202 | 123 | glassy-carbon | Angew. Chem., Int. Ed., **2018**, 57, 8614–8618 |
| **Co SAs/$Mo_2C$** | 1.0 M KOH | 178 | 155 | carbon cloth | J. Mater. Chem. A, **2020**, 8, 3071–3082 |
| **Co-BM-C** | 1.0 M KOH | 126 | 81 | Ni foam | Chem. Eng. J. **2021**, 433, 134089. |
| **Mo-$Co_9S_8$@C** | 1.0 M KOH | 113 | 67.6 | carbon paper | Adv. Energy Mater. **2020**, 10, 1903137. |
| **CoSAs-$MoS_2$/TiN NRs** | 1.0 M KOH | 131.9 | 56.9 | carbon cloth | Adv Funct Mater, **2021**, 31,2100233. |
| **NiCo DASs/N-C** | 1.0 M KOH | 189 | 72.5 | glassy-carbon | Adv. Funct. Mater. **2022**, 2210867 |
| **CoNC-SA/N*−C** | 1.0 M KOH | 194 | 91.9 | glassy-carbon | ACS Catal. **2022**, 12, 10771−10780 |
| **SC-CuSA-NC** | 1.0 M KOH | 124 | 107.5 | glassy-carbon | Composites Part B **2023**, 253, 110575 |
| **Co SAs-Co NPs/NCFs** | 1.0 M KOH | 205 | 83.2 | glassy-carbon | Journal of Energy Chemistry **2022**, 67, 147-156 |
| **120Ni-MSAC** | 1.0 M KOH | 190 | 83.5 | glassy-carbon | Chemical Engineering Journal **2023**, 468, 43733 |
| **CoNC-SA/N*−C** | 1.0 M KOH | 194 | 91.9 | glassy-carbon | ACS Catal. **2022**, 12, 10771−10780 |

**Table S6** Comparison of water splitting performance for $Ni_{SA}$-O/$Mo_2C$ and others single-atom catalysts reported in the literature at current densities of 50, 100, 100 mA $cm^{-1}$.

| Electrocatalysts | Electrolyte | 50 (mA | 100 (mA | 200 (mA | Working | Ref |
|---|---|---|---|---|---|---|

| | | cm$^{-1}$) | cm$^{-1}$) | cm$^{-1}$) | electrode | |
|---|---|---|---|---|---|---|
| **$Ni_{SA}$-O/$Mo_2C$\|\|$Ni_{SA}$-O/$Mo_2C$** | 1.0 M KOH | ~1.79 | 1.85V | 1.93 | carbon cloth | **This work** |
| **$Ir_1$@Co/NC\|\| $Ir_1$@Co/NC** | 1.0 M KOH | ~1.85 | | | carbon paper | Angew. Chem. Int. Ed., **2019**, 58,2-8. |
| **Fe-$N_4$ SAs/NPC\|\|Fe-$N_4$ SAs/NPC** | 1.0 M KOH | ~2.0 | | | carbon paper | Angew. Chem., Int. Ed., **2018**, 57, 8614–8618 |
| **CoSAs-$MoS_2$/TiN NRs** | 1.0 M KOH | 2.02 | | | carbon cloth | Adv Funct Mater., **2021**, 31,2100233. |
| **Co-BM-C\|\|Co-BM-C** | 1.0 M KOH | ~1.88 | ~1.91 | ~2.1 | Ni foam | Chem. Eng. J., **2021**,134089. |
| **Rh SAC−CuO NAs/CF \|\| Rh SAC−CuO NAs/CF** | 1.0 M KOH | ~1.65 | ~1.7 | 1.77 | copper foam | Nano letters, **2020**, 20(7): 5482-5489. |

**Table S7** Structural parameters of $Ni_{SA}$-O/$Mo_2C$ after HER-IT and OER-IT extracted from the EXAFS fitting.

| **Sample** | **Scattering pair** | **CN** | **R(Å)** | **σ $^2$($10^{-3}$Å$^2$)** | **Δ$E_0$(eV)** |
|---|---|---|---|---|---|
| **$Ni_{SA}$-O/$Mo_2C$** | Ni-O | 3.9±0.4 | 2.01±0.02 | 5.6±0.5 | 3.4±0.6 |
| | Ni-Mo | 5.0±0.8 | 2.98±0.02 | 6.9±0.6 | -5.3±0.5 |
| **HER-IT** | Ni-O | 5.9 | 2.05 | 5.9 | -0.7 |
| | Ni-Mo | 4.5 | 3.31 | 7.3 | -6.8 |
| **OER-IT** | Ni-O | 5.7 | 2.05 | 5.6 | -2.6 |
| | Ni-Ni | 1.6 | 3.07 | 5.8 | -6.1 |
| | Ni-Mo | 1.3 | 3.34 | 12.6 | -3.2 |
